# Association between vascular endothelial growth factor gene polymorphisms and the risk and prognosis of renal cell carcinoma: A systematic review and meta-analysis

**DOI:** 10.18632/oncotarget.17293

**Published:** 2017-04-20

**Authors:** Jingyuan Tang, Zhiqiang Qin, Xiao Li, Peng Han, Feng Wang, Chengdi Yang, Ran Li, Kunpeng Wang, Min Tang, Wei Wang, Qiang Lv, Wei Zhang

**Affiliations:** ^1^ Department of Urology, The First Affiliated Hospital of Nanjing Medical University, Nanjing, 210009, China; ^2^ Department of Urology, Wuxi Second People's Hospital, Nanjing Medical University, Wuxi, 214002, China; ^3^ Department of Urologic Surgery, The Affiliated Cancer Hospital of Jiangsu Province of Nanjing Medical University, Nanjing, 210029, China; ^4^ Department of Radiation Oncology, The First Affiliated Hospital of Nanjing Medical University, Nanjing, 210009, China; ^5^ Department of Urology, The First People's Hospital of Lianyungang City, Lianyungang, 222002, China

**Keywords:** VEGF polymorphisms, renal cell carcinoma, risk, prognosis, meta-analysis

## Abstract

The aim of the meta-analysis was to clarify the associations between vascular endothelial growth factor (VEGF) polymorphisms and the risk and prognosis of renal cell carcinoma (RCC). A meta-analysis was performed by searching the databases PubMed, EMBASE and Web of Science for the relevant available studies until August 1st, 2016, and fourteen studies met the inclusion criteria. The pooled odds ratios (ORs) with 95% confidence intervals (CIs) were calculated to evaluate the strength of such associations. Besides, the pooled hazard ratios (HRs) with 95% CIs were used to evaluate the overall survival (OS). Fixed- or random-effects models were conducted according to existence of heterogeneity. Publication bias was evaluated using Begg's funnel plots and Egger's regression test. Overall, this meta-analysis included a total of 8,275 patients, who had been accrued between November 2002 and September 2015. Meta-analysis indicated that -2578C/A, +936C/T and +405G/C polymorphisms in the VEGF gene correlated with elevated RCC risk, especially in Asian populations. Moreover, VEGF -1154G/A and -634C/G polymorphisms were found significantly associated with poor OS of RCC. Therefore, this meta-analysis revealed that VEGF -2578C/A, +936C/T, +405G/C polymorphisms were associated with an elevated susceptibility to RCC, indicating that these three polymorphisms might be risk factors for RCC, especially in Asian populations.

## INTRODUCTION

Renal cell carcinoma (RCC) is the most common malignancy of the kidney, accounting for approximately 80%-85% of all renal tumors [1]. Until now, many influencing factors have been identified that might increase the risk of RCC, including tobacco smoking, hypertension and occupational exposures to chemicals [2, 3]. However, even if many people are exposed to these risk factors, only a few of them develop RCC. This suggests that genetic factors may have a critical influence on the aetiology of RCC. Several studies have confirmed the role of genetic factors in the development of RCC [4, 5].

Angiogenesis is the formation of new blood vessels from endothelial precursors and it is one of the fundamental processes in carcinogenesis [6, 7]. It is well known that angiogenesis is correlated with tumor progression and metastasis [8]. Vascular endothelial growth factor (VEGF), known as a critical angiogenesis factor, could promote tumor development and progression both *in vitro* and *in vivo* experiments [9–11]. Meanwhile, tumor-induced angiogenesis and growth could be suppressed by inhibiting VEGF signaling [12, 13]. The gene encoding VEGF, which comprises a 14-kb coding region with 8 exons, is located at chromosome 6p21.3 [14]. There are many single nucleotide polymorphisms (SNPs) identified in the VEGF gene, which can alter the expression level of this gene and confer individual susceptibility to tumor [15, 16].

Compared to healthy renal tissue, the VEGF expression level is higher in RCC tissue [17]. Moreover, specific drugs targeting VEGF have shown clinical effcacy in the treatment of RCC [18, 19]. Therefore, VEGF polymorphisms may be associated with the progression and prognosis of RCC. Several SNPs, such as VEGF -2578C/A, −1156G/A, +1612G/A, +936C/T, and -634G/C, have been reported to be associated with cancer susceptibility, tumor growth and metastases in RCC patients [20–22]. However, because of the limited sample size, these studies showed the results remained inconclusive. Thus, we performed a systemic review and an updated meta-analysis including all eligible case-control studies to investigate whether VEGF polymorphisms were associated with the risk and prognosis of RCC.

## RESULTS

### Studies characteristics

**Figure 1:**
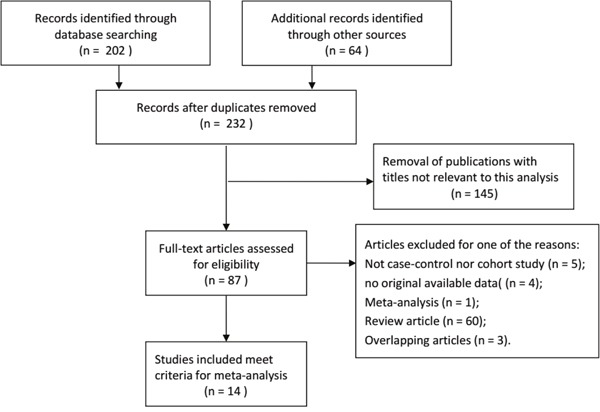
The flowchart of literature search and selection procedure

According to the searching criteria, a total of related 232 articles through a primary search of databases and reference lists were initially identified. As a result, of these articles, 14 full-text studies met the inclusion criteria and were involved in the present meta-analysis for a more further evaluation, which had been accrued between November 2002 and September 2016 [21–34]. Besides, all studies suggested that the distribution of genotypes in the controls was consistent between HWE. The flowchart of literature search and selection procedure is shown in Figure 1. In this meta-analysis, all of the baseline characteristics of the studies associated with the risk and prognosis of RCC are comprehensively listed in Table 1 and Table 2. Among all the SNPs of the VEGF gene addressed, −2578C/A, +936C/T, +1612G/A, −634G/C, +460T/C, +405 G/C, −1154G/A were the most common. Among these 14 enrolled studies, there were 10 studies based on Asian population and 4 studies conducted in Caucasians population.

**Table 1:** Baseline characteristics of studies associated with the risk of RCC included in the meta-analysis

Ref.	Year	Surname	Ethnicity	SOC	Genotyping	Cases	Controls	Gene polymorphism	NOS	HWE
[26]	2015	Yang	Asian	HB	Taqman	191	376	−2578C/A, −1154G/A, −634G/C, +936C/T	6	Y
[23]	2015	Shen	Asian	HB	PCR-RFLP	360	360	−2578C/A, +1612G/A, +936C/T, −634G/C	5	Y
[24]	2015	Xian	Asian	HB	PCR-RFLP	266	532	−2578C/A, +1612G/A, +936C/T, −634G/C	5	Y
[25]	2015	Lu	Asian	HB	PCR-RFLP	412	824	−2578C/A,+1612G/A,+460T/C, +936C/T, −634G/C	5	Y
[27]	2014	Qin	Asian	HB	Taqman	859	1004	+405G/C	7	Y
[28]	2013	Sáenz-López	Caucasian	HB	Taqman	216	280	−2578C/A, +460T/C, +405G/C, +936C/T	6	Y
[29]	2011	Ajaz	Asian	HB	PCR-RFLP	143	106	−2578C/A,+936C/T	5	Y
[30]	2010	Bruyère	Caucasian	HB	PCR-RFLP	51	202	+460T/C, +405G/C, +936C/T, −1154G/A	5	Y
[31]	2009	Ricketts	Caucasian	HB	PCR-RFLP	317	295	−1154G/A	6	Y
[32]	2002	Abe	Asian	HB	PCR-RFLP	145	145	+936C/T, +1612G/A	5	Y

SOC: source of control; HB: hospital-based; HWE: Hardy–Weinberg equilibrium.

NOS: Newcastle-Ottawa Scale

**Table 2:** Baseline characteristics of studies associated with the prognosis of RCC included in the meta-analysis

Ref.	Year	Surname	Ethnicity	Genotyping	Cases	Gene polymorphism	Survival analysis	Source of HR	Max months for follow-up	NOS
[33]	2015	Ma	Asian	PCR-RFLP	310	−2578C/A, −1154G/A, −634C/G	OS	Reported	60	7
[34]	2015	Yang	Asian	PCR-RFLP	336	−2578C/A, −1154G/A, −634C/G	OS	Reported	60	6
[21]	2014	Zhong	Asian	PCR-RFLP	332	−2578C/A, −1154G/A, −634C/G	OS	Reported	60	5
[22]	2007	Kawai	Caucasian	PCR-RFLP	213	−2578C/A, −1154G/A, −634C/G	OS	Reported	160	6

HR: hazard ratio; OS: overall survival; NOS: Newcastle-Ottawa Scale.

### Quantitative synthesis results

Overall, the strength of association between VEGF genetic polymorphisms and RCC risk was evaluated by the pooled ORs with 95% CIs based on five genetic comparison models. A summary of all the meta-analysis results for the seven studied VEGF polymorphisms and RCC susceptibility is provided in Table 3. In addition, the results of subgroup analysis by ethnicity is shown in Supplementary Table 1.

**Table 3:** Meta-analysis results for the seven studied polymorphisms and RCC risk

Genotype comparison	OR [95% CI]	Heterogeneity-test		Model
		P for Q test	I2(%)	
**VEGF -2578C/A (1588 cases, 2470 controls)**				
A vs C (Allele model)	1.30 [1.18, 1.43]	0.689	0	Fixed
AA vs CC (Homozygous model)	1.60 [1.31, 1.96]	0.430	0	Fixed
CA vs CC (Heterozygous model)	1.24 [1.08, 1.43]	0.062	52.4	Fixed
AA+CA vs CC (Dominant model)	1.31 [1.15, 1.50]	0.051	54.5	Fixed
AA vs CA+CC (Recessive model)	1.39 [1.16, 1.67]	0.626	0	Fixed
**VEGF +936C/T (1636 cases, 2712 controls)**				
T vs C (Allele model)	1.16 [1.05,1.29]	0.130	39.3	Fixed
TT vs CC (Homozygous model)	1.33 [1.08, 1.65]	0.236	25.3	Fixed
CT vs CC (Heterozygous model)	1.13 [0.97, 1.30]	0.246	23.9	Fixed
TT+CT vs CC (Dominant model)	1.16 [1.02, 1.33]	0.123	40.2	Fixed
TT vs CT+CC (Recessive model)	1.25 [1.02, 1.52]	0.478	0	Fixed
**VEGF +1612G/A (1184 cases, 1862 controls)**				
A vs G (Allele model)	1.08 [1.00,1.17]	0.639	0	Fixed
AA vs GG (Homozygous model)	1.33 [1.02, 1.74]	0.527	0	Fixed
GA vs GG (Heterozygous model)	1.09 [0.93, 1.27]	0.919	0	Fixed
AA+GA vs GG (Dominant model)	1.12 [0.96, 1.30]	0.760	0	Fixed
AA vs GA+GG (Recessive model)	1.27 [0.99, 1.64]	0.558	0	Fixed
**VEGF -634G/C (1229 cases, 2092 controls)**				
C vs G (Allele model)	1.11 [1.00,1.23]	0.882	0	Fixed
CC vs GG (Homozygous model)	1.22 [0.99, 1.51]	0.964	0	Fixed
GC vs GG (Heterozygous model)	1.13 [0.96, 1.32]	0.998	0	Fixed
CC+GC vs GG (Dominant model)	1.15 [0.99, 1.33]	0.994	0	Fixed
CC vs GC+GG (Recessive model)	1.14 [0.94, 1.38]	0.961	0	Fixed
**VEGF +460T/C (677 cases, 1299 controls)**				
C vs T (Allele model)	0.92 [0.58,1.46]	0.000	87.9	Random
CC vs TT (Homozygous model)	0.88 [0.38, 2.01]	0.006	80.6	Random
TC vs TT (Heterozygous model)	1.12 [0.89, 1.41]	0.235	31	Fixed
CC+TC vs TT (Dominant model)	0.98 [0.61, 1.58]	0.017	75.5	Random
CC vs TC+TT (Recessive model)	1.10 [0.87, 1.39]	0.011	77.9	Random
**VEGF +405 G/C (1086 cases, 1460 controls)**				
C vs G (Allele model)	1.18 [1.05,1.33]	0.113	54.1	Fixed
CC vs GG (Homozygous model)	1.35 [1.06,1.72]	0.125	51.8	Fixed
GC vs GG (Heterozygous model)	1.25 [1.05,1.48]	0.407	0	Fixed
CC+GC vs GG (Dominant model)	1.27 [1.08,1.49]	0.191	39.5	Fixed
CC vs GC+GG (Recessive model)	1.06 [0.84,1.33]	0.628	0	Fixed
**VEGF -1154G/A (564 cases, 892 controls)**				
A vs G (Allele model)	1.04 [0.88,1.24]	0.228	32.3	Fixed
AA vs GG (Homozygous model)	1.07 [0.73,1.57]	0.330	9.7	Fixed
GA vs GG (Heterozygous model)	1.06 [0.84,1.35]	0.392	0	Fixed
AA+GA vs GG (Dominant model)	1.06 [0.84,1.32]	0.266	24.4	Fixed
AA vs GA+GG (Recessive model)	1.04 [0.72,1.51]	0.493	0	Fixed

### -2578C/A and RCC risk

**Figure 2:**
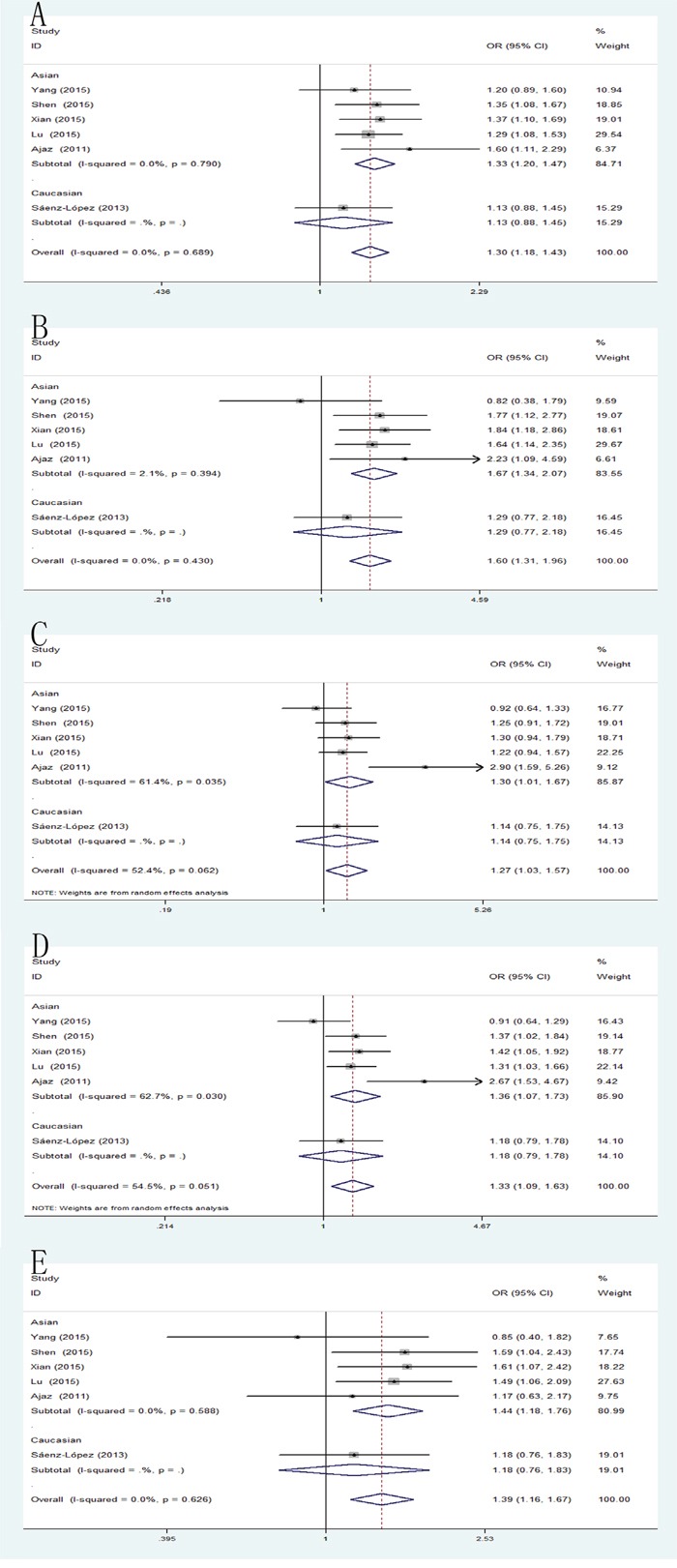
Forest plot of the association between the -2578C/A polymorphism and RCC risk **(A)** allele model; **(B)** homozygote model; **(C)** heterozygote model; **(D)** dominant model; **(E)** recessive model.

The combined results of all analyses showed that the pooled OR of these six studies was 1.30 (95% CI: 1.18-1.43) for allele model, 1.60 (95% CI: 1.31-1.96) for homozygote model, 1.24 (95% CI: 1.08-1.43) for heterozygote model, 1.31 (95% CI: 1.15-1.50) for dominant model and 1.39 (95% CI: 1.16-1.67) for recessive model, which indicated a strong association between VEGF -2578C/A mutation and RCC susceptibility. Furthermore, subgroups analysis by ethnicity was performed to establish the effects of heterogeneity on the results. In the subgroup analysis by ethnicity, the results were significant in Asian populations rather than Caucasians population in all genetic models, including allele model: pooled OR=1.33, 95% CI: 1.20-1.47; homozygote model: pooled OR=1.67, 95% CI: 1.34-2.07; heterozygote model: pooled OR=1.30, 95% CI: 1.01-1.67; dominant model: pooled OR=1.36, 95% CI: 1.07-1.73; recessive model: pooled OR=1.44, 95% CI: 1.18-1.76 (Figure 2).

### +936C/T and RCC risk

**Figure 3:**
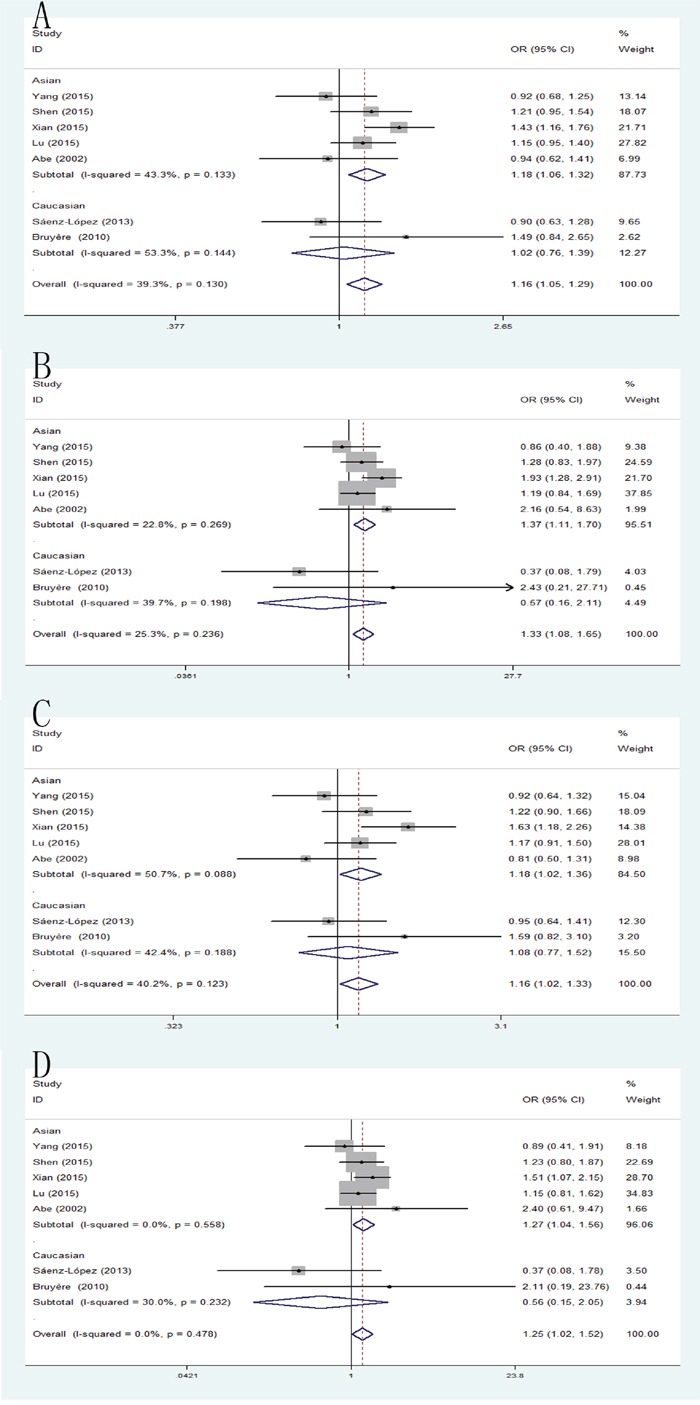
Forest plot of the association between the +936C/A polymorphism and RCC risk **(A)** allele model; **(B)** homozygote model; **(C)** dominant model; **(D)** recessive model.

Significant differences were found between VEGF +936C/T polymorphism and RCC risk using allele model: pooled OR=1.16, 95% CI: 1.05-1.29; homozygote model: pooled OR=1.33, 95% CI: 1.08-1.65; dominant model: pooled OR=1.16, 95% CI: 1.02-1.33; recessive model: pooled OR=1.25, 95% CI: 1.02-1.52. When the studies were stratified by ethnicity, significant differences were observed in Asian population in those models (allele model: pooled OR=1.18, 95% CI: 1.06-1.32; homozygote model: pooled OR=1.37, 95% CI: 1.11-1.70; dominant model: pooled OR=1.18, 95% CI: 1.02-1.36; recessive model: pooled OR=1.27, 95% CI: 1.04-1.56), but, no significant results were detected in Caucasian population (Figure 3).

### +1612G/A and RCC risk

**Figure 4:**
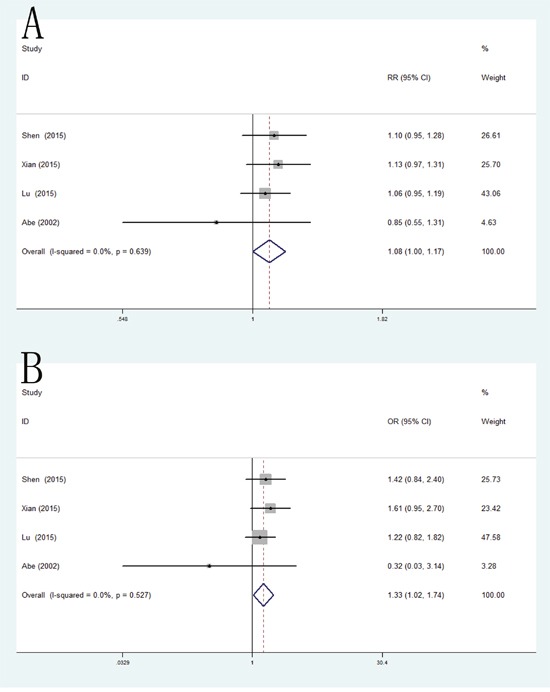
Forest plot of the association between the +1612C/A polymorphism and RCC risk **(A)** allele model; **(B)** dominant model.

In the present meta-analysis, the results demonstrated that the VEGF +1612G/A polymorphism was significant correlated with RCC only in allele model (pooled OR = 1.08, 95% CI: 1.00-1.17) and homozygote model (pooled OR = 1.33, 95% CI: 1.02-1.74) (Figure 4).

### -634G/C and RCC risk

No statistically significant association between VEGF -634G/C polymorphism and RCC susceptibility was assessed in all genetic comparison models and the stratified analysis by ethnicity.

### +460T/C and RCC risk

**Figure 5:**
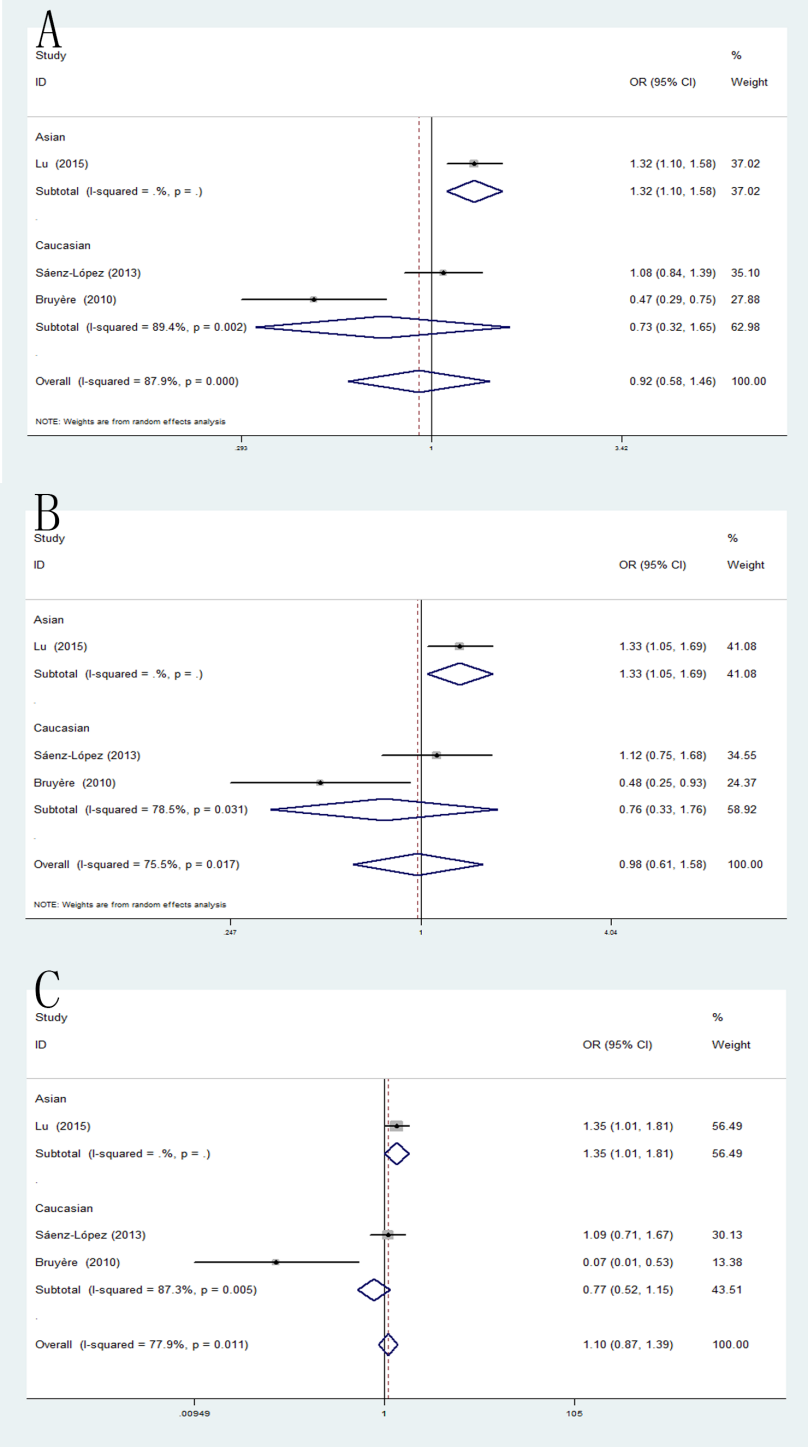
Forest plot of the association between the +460C/A polymorphism and RCC risk **(A)** allele model; **(B)** homozygote model; **(C)** recessive model.

The pooled analysis has shown that the VEGF +460T/C polymorphism was not significantly associated with RCC susceptibility in all five genetic models. However, when the studies were stratified by ethnicity, the positive result was detected in Asian population for allele model (pooled OR = 1.32, 95% CI: 1.10-1.58), dominant model (pooled OR = 1.33, 95% CI: 1.05-1.69) and recessive model (pooled OR = 1.35, 95% CI: 1.01-1.81) (Figure 5).

### +405 G/C and RCC risk

**Figure 6:**
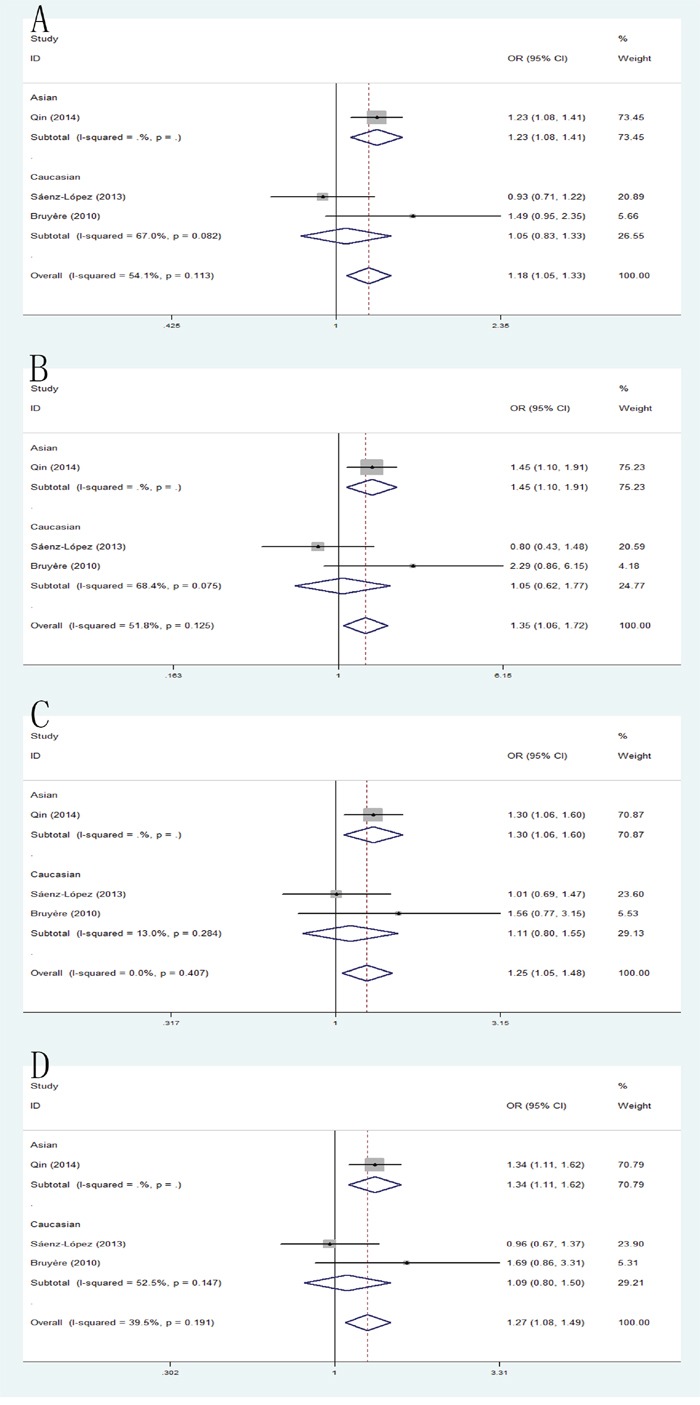
Forest plot of the association between the +405C/A polymorphism and RCC risk **(A)** allele model; **(B)** homozygote model; **(C)** heterozygote model; **(D)** dominant model.

The combined results of all analyses showed that the pooled OR of these studies was 1.18 (95% CI: 1.05-1.33) for allele model, 1.35 (95% CI: 1.06-1.72) for homozygote model and 1.25 (95% CI: 1.05-1.48) for heterozygote model and 1.27(95% CI: 1.08-1.49) for dominant model, which indicated a significant association between VEGF +405 G/C polymorphism and RCC risk. In addition, when the studies were stratified by ethnicity, the significant results were found only in Asian populations in allele model: pooled OR=1.23, 95% CI: 1.08-1.41; homozygote model: pooled OR=1.46, 95% CI: 1.10-1.91; heterozygote model: pooled OR=1.30, 95% CI: 1.06-1.60; dominant model: pooled OR=1.34, 95% CI: 1.11-1.62 (Figure 6).

### -1154G/A and RCC risk

In all genetic models, no significant differences was found in VEGF -1154G/A mutation for RCC risk.

### VEGF genetic polymorphisms and prognosis of RCC

**Figure 7:**
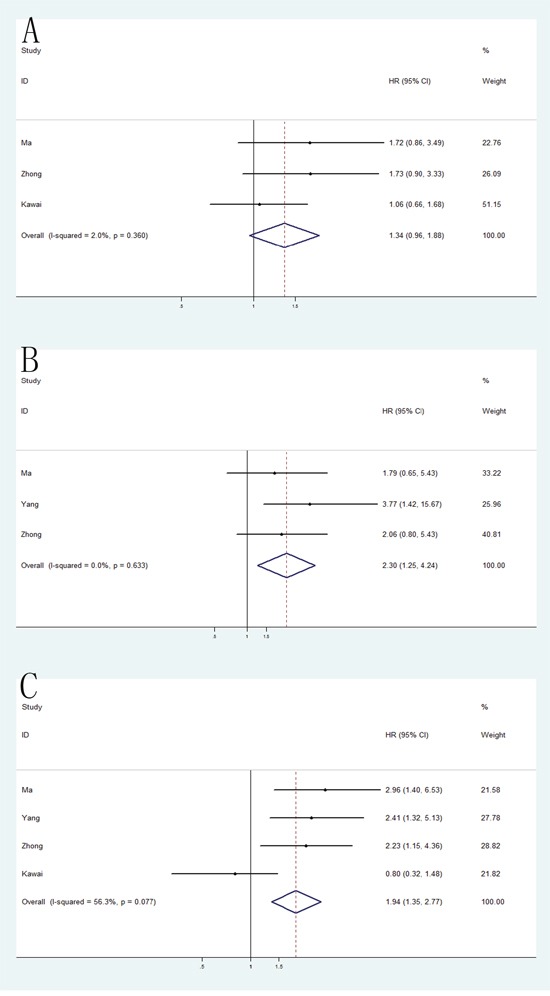
Forest plot of the association between -2578C/A, −1154G/A and -634C/G polymorphism and the overall survival of RCC

Because of the large number of studies evaluating the relationship of three individual VEGF polymorphisms (VEGF -2578C/A, −1154G/A and -634C/G; individual details in Table 2) and the prognosis of RCC, meta-analyses were performed separately on these polymorphisms. As a result, VEGF -1154G/A and -634C/G polymorphisms were found significantly associated with poor OS in homozygote model (−1154G/A: HR = 2.30, 95% CI = 1.25-4.24; -634C/G: HR = 1.94, 95% CI = 1.35-2.77). However, no significant differences was detected in VEGF -2578C/A polymorphism (HR = 1.34, 95% CI = 0.96-1.88) (Figure 7).

### Sensitivity analysis

Individual studies were consecutively omitted in the sensitivity analysis to detect the influence of each study on the pooled OR. The sensitivity analysis for the results of VEGF genetic polymorphisms and RCC risk demonstrated that the obtained results were statistically robust and no individual study affected the pooled OR significantly.

### Publication bias

**Figure 8:**
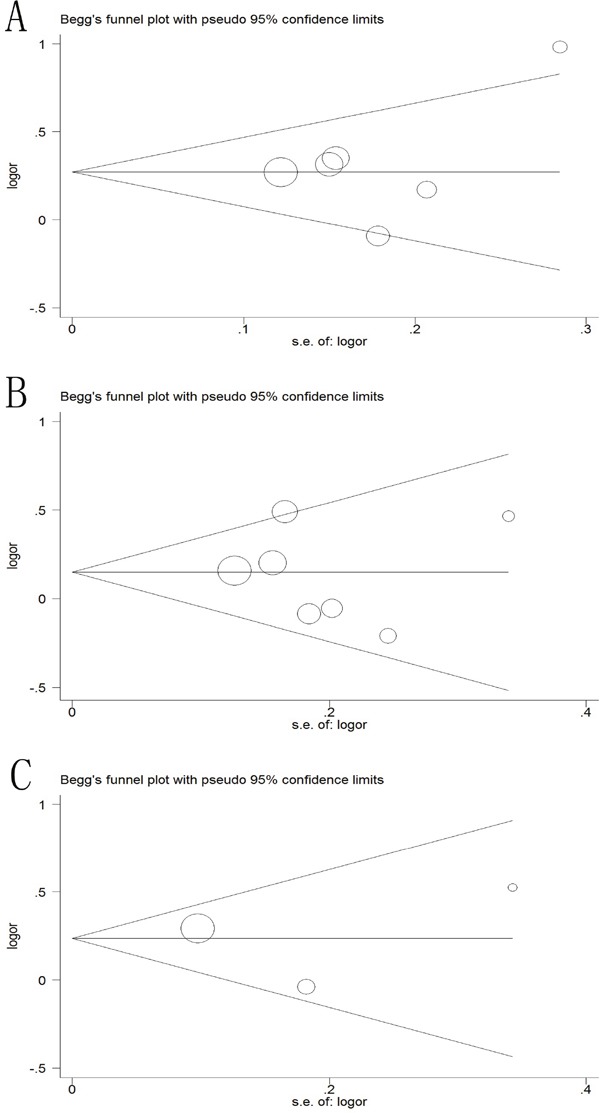
Begg's funnel plot of publication bias test in the dominant model **(A)** -2578C/A polymorphism; **(B)** +936C/A polymorphism; **(C)** +405C/A polymorphism.

The Begg's funnel plot and Egger's test were applied to assess the publication bias of the literature in the meta-analysis. As illustrated in Figure 8, the shapes of funnel plots showed no evidence of obviously asymmetrical, indicating no evidence of publication bias in dominant model (−2578C/A, *P*=0.573; +936C/T, *P*=0.652; +405 G/C, *P*= 0.602). Moreover, no publication bias was found in test for -2578C/A, −1154G/A, −634C/G on survival. Therefore, our results were reliable according to the overall findings.

## DISCUSSION

As a multifunctional cytokine, VEGF plays a crucial role in the regulation of angiogenesis which has attracted attention because of its involvement in the development of some common cancers [8, 35]. VEGF could promote endothelial cell proliferation and the development of new blood vessels [36]. The process of carcinogenesis might be accelerated when the VEGF gene expression was influenced by some functional gene variations. Moreover, the serum VEGF levels from cancer patients were significantly higher than those without signs of cancer [37]. There are several polymorphisms in the VEGF gene and previous studies have indicated many polymorphisms are correlated with various types of cancer including gastric, lung, prostate, breast and renal cancer [38–40]. VEGF gene polymorphisms could influence the susceptibility, tumor grade and OS of cancer [22, 41].

Renal cancer is one of the most common cancers, and it is a major clinical and societal problem in the world [1]. There is growing evidence that aberrations in VEGF contribute to RCC [42]. Moreover, RCC accounts for 2-3% of human cancer and is mainly attributed to the frequent mutations of the von HippelLindau (VHL) tumor suppressor gene [43]. During the past several years, many case-control studies published have assessed the associations of VEGF polymorphisms with the risk of renal cancer, but the findings were inconsistent [23–26]. A case-control study in a Chinese population indicated that individuals with the AA genotype and A allele of -2578C/A significantly increased the risk of RCC, when compared with the CC genotype [24]. However, another case-control study showed -2578C/A polymorphism did not appear to exert a significant influence on the risk of RCC [28].

A recent meta-analysis suggested that VEGF +936C/T, +1612G/A, −1154G/A, −2549I/D, −460T/C and +405G/C gene polymorphisms were not associated with the risk of RCC, while -2578C/A gene polymorphism might increase the risk under specific genetic models [44]. However, the author only reviewed 5 studies. Another meta-analysis showed that the +936C/T and -2578C/A polymorphisms of VEGF were associated with an increased risk for renal cell carcinoma [45]. However, none of the two meta-analysis found the significant association between VEGF +405G/C gene polymorphisms and RCC risk due to limited published studies, and the latter meta-analysis did not perform the subgroup analysis. Thus, the conclusion in the previous studies was still inaccurate. In addition, those authors failed to assess the predictive value of VEGF polymorphisms in the prognosis of RCC. In recent years, more studies have evaluated the connection between VEGF polymorphisms and the risk and prognosis of RCC [23–27]. To the best of our knowledge, the sample size in the meta-analysis is larger than any individual study, making more precise and robust results. Thus, the present meta-analysis aimed to provide a more powerful and reliable conclusion about the relationship between VEGF genetic polymorphisms and RCC susceptibility.

This meta-analysis of individual patient data showed that VEGF -2578C/A, +936C/T, +405G/C polymorphisms correlated with an elevated risk of RCC, indicating that these polymorphisms might be risk factors for RCC, especially in Asian populations. However, no statistically significant association was found between -634G/C, −1154G/A, +1612G/A polymorphisms and RCC risk. Interestingly, VEGF +460T/C polymorphism was found significantly associated with susceptibility of RCC, only in Asian populations. Though the exact mechanism of ethnic differences was unknown, a possible reason could be genetic drift and natural selection [46].

To investigate the association between VEGF variants and the prognosis of RCC, 4 independent case-control studies were included with a total of 1,191 RCC cases, the results revealed that significant differences were found in VEGF -1154G/A and -634C/G polymorphisms. Nevertheless, VEGF -2578C/A mutation was not significantly associated with poor OS. The discrepancy is likely that -2578C/A is involved in RCC initiation, and VEGF -1154G/A and -634C/G are involved in progression or treatment response, or tumor heterogeneity [47].

To a certain extent, some limitations should also be emphasized when interpreting the data. (1) Most populations included in this meta-analysis were Asian and Caucasian ethnicity, and more populations from other ethnicities will be required in the future research. (2) The number of included studies in some subgroups was relatively small, with limited statistical power to investigate the real association. More studies by standardized unbiased methods are required, which can offer more detailed individual data of high quality. (3) Adjusted estimates could not be performed in our meta-analysis without enough data for the adjustment by other RCC covariates such as age, life-style and so on. (4) No available data assessing the association between VEGF genetic polymorphisms and the OS of RCC was obtained in some included studies. Therefore, further high-quality researches in RCC prognosis might be performed to draw more accuracy results in subsequent years.

## CONCLUSION

In summary, the results of the current meta-analysis indicated that VEGF -2578C/A, +936C/T, +405 G/C polymorphisms were associated with an elevated susceptibility to RCC, indicating that these three polymorphisms might be risk factors for RCC, especially in Asian populations. Moreover, VEGF -1154G/A and -634C/G polymorphisms were found significantly associated with poor prognosis of RCC and might become a predicted biomarker in the future. Additional high-quality and multicenter studies with larger sample sizes are needed to confirm our findings in subsequent articles.

## MATERIALS AND METHODS

According to the Preferred Reporting Items for Systematic Reviews and Meta-Analyses (PRISMA), we performed a comprehensive search based on PubMed, EMBASE and Web of Science obtain relevant studies published until August 1st, 2016. The combination of the following search items and MeSH terms were utilized: “vascular endothelial growth factor” or “VEGF”, “polymorphism” or “mutation”, “variants” and “renal cell carcinoma” or “kidney cancer”. Other potentially eligible literature were collected by manually searching from relevant reviews and the references of original studies included for the meta-analysis. Besides, all remaining articles were checked to prevent overlapping datasets. Furthermore, because the data was from previously published studies, ethical approval and informed consent were not required.

Eligible studies were selected according to the following inclusion criteria: (1) Independent case-control or cohort studies; (2) The association between VEGF variants and the risk and the prognosis of RCC; (3) Inclusion of adequate data on frequency of genotypes including ORs and the their 95% CIs. In addition, the study did not meet the inclusion criteria was excluded.

### Data extraction

According to the above the inclusion criteria, two investigators (Tang JY and Qin ZQ) independently extracted available data from the eligible studies identified, and any disagreements were resolved by discussion with a third reviewer until a consensus was reached. All the extracted information were recorded in a standardized form and the extracted elements included: year of publication, first author's last name, ethnicity, source of controls, genotyping assay, the number of cases and controls, the number of VEGF gene polymorphisms carriers and non-carriers respectively, and the results of the Hardy-Weinberg equilibrium (HWE) test.

### Quality assessment

The quality of the studies was assessed using the validated Newcastle-Ottawa Scale (NOS) for nonrandomized studies. A study can be awarded a maximum of one star for each point within the selection and exposure categories, and a maximum of two stars can be given for comparability. We considered studies with scores of more than 5 as high-quality studies and only high-quality studies were included in our meta-analysis.

### Statistical analysis

The strength of association between VEGF mutations and RCC susceptibility was evaluated by the pooled odds ratios (ORs) with 95% confidence intervals (CIs) based on five genetic comparison models: allele model, homozygous model, heterozygous model, dominant model and recessive model. For the primary assessment of the association between VEGF polymorphisms and the overall survival (OS) of RCC, we used the overall hazard ratios (HRs). The goodness-of-fit chi-square test was adopted to assess HWE in controls and P<0.05 was regarded as significant disequilibrium. The pooled ORs were calculated either with fixed-effects model (a Mantel-Haenszel method) or with the random-effects model (a DerSimonian-Laird method) according to the *P* values of study heterogeneities. If there was no indication of substantial heterogeneity, the fixed-effects model would be conducted. Otherwise, the random effects model was selected to perform this meta-analysis. Then subgroup analysis according to ethnicity was further carried out to explore the potential sources of heterogeneity. To examine the stability and reliability of the overall meta-analysis results, sensitivity analysis was performed by excluding one single study one by one and recalculating their ORs. In addition, Begg's funnel plots and Egger's linear regression test were employed to search for publication bias between the studies, and *P* values were deemed as a significantly selective bias when less than 0.05. STATA software (version 12.0; StataCorp LP, College Station, TX) was utilized to deal with all above statistical analyses.

## SUPPLEMENTARY MATERIALS TABLE




