# Supplementary material for: Association between vascular endothelial growth factor gene polymorphisms and the risk and prognosis of renal cell carcinoma: A systematic review and meta-analysis
Source: Oncotarget. 2017 Apr 20;8(30):50034–50. doi: 10.18632/oncotarget.17293 (PMC5564826; doi:10.18632/oncotarget.17293)
Supplement: Supplementary file 1 [file oncotarget-08-50034-s001.pdf]

Association between vascular endothelial growth factor gene polymorphisms and the risk and prognosis of renal cell carcinoma: A systematic review and meta-analysis

SUPPLEMENTARY MATERIALS

SUPPLEMENTARY TABLE

Supplementary Table 1: Results of subgroup analysis by ethnicity in the association between VEGF polymorphisms and RCC susceptibility

See Supplementary File 1
