# Supplementary material for: Association between vascular endothelial growth factor gene polymorphisms and the risk and prognosis of renal cell carcinoma: A systematic review and meta-analysis
Source: Oncotarget. 2017 Apr 20;8(30):50034–50. doi: 10.18632/oncotarget.17293 (PMC5564826; doi:10.18632/oncotarget.17293)
Supplement: Supplementary file 2 [file oncotarget-08-50034-s002.doc]

| **Table S1:Results of subgroup analysis** | | | | | | | | |
| --- | --- | --- | --- | --- | --- | --- | --- | --- |
| **Genotype comparison** | | **Subgroup** | **OR [95% CI]** | **Heterogeneity-test** | | | | **Model** |
|  |  |  |  | P for Q test | | | I2(%) |  |
| **VEGF -2578C/A** | | | | | | | | |
| A vs C (Allele model) | | Asian | 1.33 [1.20, 1.47] | | 0.79 | | 0 | Fixed |
|  |  | Caucasian | 1.13 [0.88, 1.45] | | - | | - | Fixed |
| AA vs CC (Homozygous model) | | Asian | 1.67 [1.34, 2.07] | | 0.394 | | 2.1 | Fixed |
|  |  | Caucasian | 1.29 [0.77, 2.18] | | - | | - | Fixed |
| CA vs CC (Heterozygous model) | | Asian | 1.30 [1.01, 1.67] | | 0.035 | | 61.4 | Random |
|  |  | Caucasian | 1.14 [0.75, 1.75] | | - | | - | Random |
| AA+CA vs CC (Dominant model) | | Asian | 1.36 [1.07, 1.73] | | 0.03 | | 62.7 | Random |
|  |  | Caucasian | 1.18 [0.79, 1.78] | | - | | - | Random |
| AA vs CA+CC (Recessive model) | | Asian | 1.44 [1.18, 1.76] | | 0.588 | | 0 | Fixed |
|  |  | Caucasian | 1.18 [0.76, 1.83] | | - | | - | Fixed |
| **VEGF +936C/T** | | | | | | | | |
| T vs C (Allele model) | | Asian | 1.18 [1.06, 1.32] | | 0.133 | 43.3 | | Fixed |
|  |  | Caucasian | 1.02 [0.76, 1.39] | | 0.144 | 53.3 | | Fixed |
| TT vs CC (Homozygous model) | | Asian | 1.37 [1.11, 1.70] | | 0.269 | 22.8 | | Fixed |
|  |  | Caucasian | 0.57[0.16, 2.11] | | 0.198 | 39.7 | | Fixed |
| CT vs CC (Heterozygous model) | | Asian | 1.13 [0.96, 1.33] | | 0.154 | 40.1 | | Fixed |
|  |  | Caucasian | 1.12 [0.79, 1.59] | | 0.27 | 17.6 | | Fixed |
| TT+CT vs CC (Dominant model) | | Asian | 1.18 [1.02, 1.36] | | 0.088 | 50.7 | | Fixed |
|  |  | Caucasian | 1.08 [0.77, 1.52] | | 0.188 | 42.4 | | Fixed |
| TT vs CT+CC (Recessive model) | | Asian | 1.27 [1.04, 1.56] | | 0.558 | 0 | | Fixed |
|  |  | Caucasian | 0.56[0.15, 2.05] | | 0.232 | 30 | | Fixed |
| **VEGF +460T/C** | | | | | | | | |
| C vs T (Allele model) | | Asian | 1.32 [1.10, 1.58] | | - | - | | Random |
|  |  | Caucasian | 0.73 [0.32, 1.65] | | 0.002 | 89.4 | | Random |
| CC vs TT (Homozygous model) | | Asian | 1.25 [0.93, 1.68] | | - | - | | Random |
|  |  | Caucasian | 0.96 [0.67, 1.37] | | 0.201 | 38.9 | | Fixed |
| TC vs TT (Heterozygous model) | | Asian | 1.12 [0.94, 1.31] | | - | - | | Fixed |
|  |  | Caucasian | 0.83 [0.59, 1.69] | | 0.264 | 18.5 | | Fixed |
| CC+TC vs TT (Dominant model) | | Asian | 1.33 [1.05, 1.69] | | - | - | | Fixed |
|  |  | Caucasian | 0.76 [0.33, 1.76] | | 0.031 | 78.5 | | Random |
| CC vs TC+TT (Recessive model) | | Asian | 1.35 [1.01, 1.81] | | - | - | | Random |
|  |  | Caucasian | 0.77 [0.52, 1.15] | | 0.005 | 87.3 | | Random |
| **VEGF +405 G/C** | | | | | | | | |
| C vs G (Allele model) | Asian | | 1.23 [1.08, 1.41] | | - | - | | Random |
|  | Caucasian | | 1.05 [0.83, 1.33] | | 0.082 | 67 | | Random |
| CC vs GG (Homozygous model) | Asian | | 1.45 [1.10, 1.91] | | - | - | | Random |
|  | Caucasian | | 1.05 [0.62, 1.77] | | 0.075 | 68.4 | | Random |
| GC vs GG (Heterozygous model) | Asian | | 1.30 [1.06, 1.60] | | - | - | | Fixed |
|  | Caucasian | | 1.11 [0.80, 1.55] | | 0.284 | 13 | | Fixed |
| CC+GC vs GG (Dominant model) | Asian | | 1.34 [1.11, 1.62] | | - | - | | Fixed |
|  | Caucasian | | 1.09 [0.80, 1.50] | | 0.147 | 52.5 | | Fixed |
| CC vs GC+GG (Recessive model) | Asian | | 1.08 [0.84, 1.40] | | - | - | | Fixed |
|  | Caucasian | | 0.96 [0.58, 1.60] | | 0.377 | 0 | | Fixed |
| **VEGF -1154G/A** | | | | | | | | |
| A vs G (Allele model) | Asian | | 0.94 [0.68, 1.29] | | - | - | | Fixed |
|  | Caucasian | | 1.09 [0.89, 1.34] | | 0.127 | 57.1 | | Fixed |
| AA vs GG (Homozygous model) | Asian | | 0.74 [0.32, 1.73] | | - | - | | Fixed |
|  | Caucasian | | 1.19 [0.77, 1.84] | | 0.264 | 19.9 | | Fixed |
| GA vs GG (Heterozygous model) | Asian | | 1.03 [0.69, 1.53] | | - | - | | Fixed |
|  | Caucasian | | 1.08 [0.80, 1.46] | | 0.176 | 45.3 | | Fixed |
| AA+GA vs GG (Dominant model) | Asian | | 0.98 [0.67, 1.42] | | - | - | | Fixed |
|  | Caucasian | | 1.10 [0.83, 1.46] | | 0.122 | 58.1 | | Fixed |
| AA vs GA+GG (Recessive model) | Asian | | 0.74 [0.32, 1.70] | | - | - | | Fixed |
|  | Caucasian | | 1.14 [0.76, 1.73] | | 0.453 | 0 | | Fixed |
